# Biomarker-guided IL-5 blockade achieves steroid-free MPO-ANCA seroconversion in EGPA

**DOI:** 10.1016/j.jacig.2025.100580

**Published:** 2025-10-10

**Authors:** Osamu Matsuno, Tansri Wibowo, Yutaka Ishida, Atsuhsi Ogata

**Affiliations:** Department of Allergic and Rheumatoid Disease, Osaka Habikino Medical Center, 3-7-1 Habikino, Osaka, Japan

**Keywords:** EGPA, MPO-ANCA, mepolizumab, IL-5 blockade, eosinophilic vasculitis

## Abstract

This report describes a case of biopsy-confirmed EGPA in which early initiation of mepolizumab, guided by biomarker surveillance, resulted in steroid-free MPO-ANCA seroconversion. These findings suggest a potential steroid-sparing strategy for ANCA-positive EGPA.

This case report illustrates the fact that biomarker-guided biologic intervention may achieve steroid-free remission in selected patients with biopsy-proven eosinophilic granulomatosis with polyangiitis (EGPA). Gradual increases in both peripheral blood titers of eosinophils and myeloperoxidase (MPO)–antineutrophil cytoplasmic antibody (ANCA), which were detected despite the absence of clinical symptoms, prompted early administration of mepolizumab at the full EGPA-approved dose (300 mg every 4 weeks), resulting in complete serologic remission without relapse. In contrast to a recent report of severe EGPA flare during treatment with low-dose mepolizumab (100 mg every 4 weeks) plus corticosteroids, our case suggests that serial biomarker monitoring and timely dose escalation may represent a proactive strategy to prevent disease progression. This case suggests a potential role for biomarker-guided biologic modulation to prevent relapse in ANCA-positive EGPA. Although promising, this anticipatory approach requires further study before it can be generalized.

We report a case of biopsy-proven EGPA in which asymptomatic reelevation of MPO-ANCA and eosinophils after steroid tapering was successfully managed with mepolizumab. The patient initially responded favorably to systemic corticosteroids, achieving remission during the active phase of disease. This case report may support a strategy of early biologic intervention based on biomarker surveillance to prevent overt relapse in patients with ANCA-positive EGPA, although further validation is needed.

EGPA is a rare eosinophil-associated vasculitis characterized by asthma and eosinophilia, with MPO-ANCA positivity observed in a subset of patients. A longitudinal cohort study reported MPO-ANCA positivity in approximately 24% of cases.^1^ Although corticosteroids remain the cornerstone of treatment, the capacity of IL-5 blockade to independently suppress ANCA production remains uncertain.

We report a case of EGPA in which complete MPO-ANCA seroconversion was achieved with mepolizumab monotherapy following corticosteroid withdrawal. This report focuses on the long-term, steroid-free follow-up phase of management of a patient who was previously described during failure of benralizumab monotherapy in the acute phase of disease.^2^

A 58-year-old man with a 12-year history of asthma and eosinophilic chronic rhinosinusitis presented with purpuric skin eruptions, peripheral neuropathy, marked peripheral blood eosinophilia (13,680 eosinophils/μL), and radiographic findings consistent with eosinophilic pneumonia. The patient’s MPO-ANCA level was mildly elevated (10.8 IU/mL; reference value < 3.5 IU/mL). Because of prior pneumonectomy for tuberculosis, he declined corticosteroids. Benralizumab (30 mg every 4 weeks) was administered but failed to prevent disease progression, which included worsening purpura, neuropathy, and an increase in MPO-ANCA levels to 130 IU/mL. Skin biopsy confirmed necrotizing eosinophilic vasculitis. According to the 2022 American College of Rheumatology classification, the patient fulfilled 6 of the following 7 criteria for EGPA^3^: obstructive airway disease, nasal polyps, blood eosinophilia (eosinophil count ≥ 1000/μL), eosinophilic infiltration on biopsy, absence of cytoplasmic ANCA, and absence of hematuria. The remaining criterion, mononeuritis multiplex, was not present in this patient. Administration of high-dose prednisone (60 mg per day) was initiated, leading to rapid resolution of symptoms and normalization of laboratory values. The dose was gradually tapered over 12 months and subsequently discontinued.

Routine surveillance performed in the patient at age 60 years (ie, approximately 20 months after steroid cessation) revealed a rise in his eosinophil counts from 270 to 1218/μL and an increase in his MPO-ANCA titers from 0.5 to 35 IU/mL despite the absence of clinical symptoms. Concerned about subclinical disease reactivation, we initiated mepolizumab monotherapy (300 mg every 4 weeks). As shown in Fig 1, the patient’s eosinophil counts and MPO-ANCA titers both normalized within 3 months. MPO-ANCA became undetectable, and the patient has remained in sustained clinical and serologic remission for more than 12 months without corticosteroids or other immunosuppressants. The patient had previously undergone a left pneumonectomy because of tuberculosis and expressed significant concern about potential reactivation in the remaining lung. Although high-dose corticosteroids were necessary during initial disease onset owing to a high level of EGPA activity, the patient was apprehensive about resuming systemic steroids during the MPO-ANCA reelevation phase. Given his preference to avoid further steroid exposure and the absence of clinical symptoms, early intervention with mepolizumab was selected as a steroid-sparing strategy.

**Fig 1:**
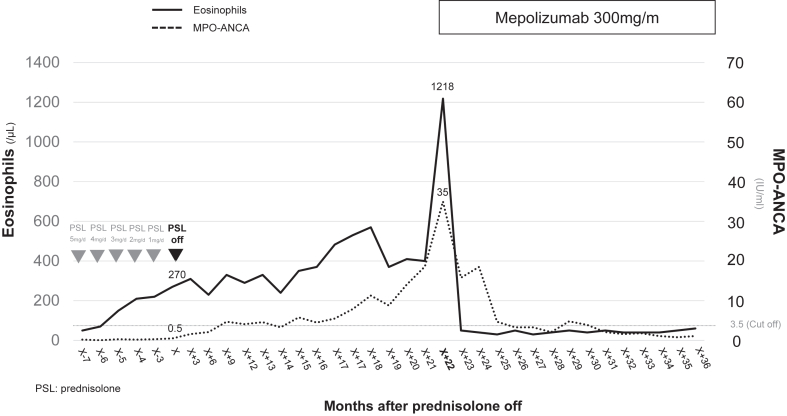
Temporal profile of peripheral eosinophil counts and MPO-ANCA titers during corticosteroid tapering and after withdrawal, followed by mepolizumab monotherapy. Blood eosinophil and MPO-ANCA levels were stable during low-dose corticosteroid tapering (from 5 mg per day to 0 mg per day). After complete steroid cessation, both markers gradually increased over a 20-month period without overt symptoms. Mepolizumab monotherapy (300 mg per month) induced rapid normalization of biomarkers within 3 months, with sustained clinical and serologic remission thereafter. The assay's cutoff value for MPO-ANCA positivity is 3.5 IU/mL.

This case provides rare clinical evidence of complete MPO-ANCA seroconversion achieved solely through IL-5 blockade. Prior reports of ANCA reduction with IL-5–targeted therapy have generally involved concurrent corticosteroid use.^4^, ^5^, ^6^ For example, benralizumab has been reported to reduce MPO-ANCA in a patient with EGPA who was receiving high-dose prednisolone and azathioprine for asthma control.^7^

In contrast, our case involved biomarker-guided initiation of mepolizumab monotherapy in a corticosteroid-free state, resulting in achievement of complete seroconversion and isolation of the effect of mepolizumab, thus suggesting a direct immunomodulatory mechanism beyond eosinophil suppression.

Although eosinophil extracellular trap cell death was not directly evaluated, the rapid decline in MPO-ANCA levels following IL-5 blockade supports a plausible link between eosinophil activity and ANCA production. This observation aligns with mechanistic models of EGPA pathogenesis involving eosinophil-driven autoantigen exposure. Importantly, Mukherjee et al demonstrated that sputum from patients who were serum ANCA–negativity with EGPA contains functionally active ANCA capable of inducing extracellular trap formation by neutrophils and eosinophils.^8^ These findings support a role for local eosinophilic inflammation in sustaining ANCA-mediated autoimmunity that is potentially modifiable through IL-5 inhibition.

Notably, this outcome was achieved in a corticosteroid-free setting, with mepolizumab initiated based on subclinical biomarker trends, suggesting a potential paradigm shift toward preemptive, biomarker-guided management of EGPA.

In the case described by Vanthuyne et al,^9^ the patient was undergoing maintenance therapy with low-dose mepolizumab (100 mg every 4 weeks) and corticosteroids. Although peripheral eosinophilia remained suppressed until clinical relapse, that patient’s MPO-ANCA levels had already begun to rise before symptom onset. In contrast, our patient was not taking any immunosuppressants, and his MPO-ANCA levels and eosinophil counts both showed gradual elevation during asymptomatic follow-up. The simultaneous increase in these markers, which was previously shown to correlate positively in EGPA,^1^ may represent a high-risk state warranting early biologic intervention.

Notably, benralizumab had previously failed to induce remission when administered during the acute phase of EGPA, shortly after symptom onset. In contrast, mepolizumab was initiated in a symptom-free state based on rising serologic markers. The difference in disease activity and timing of intervention may partly explain the contrasting outcomes, highlighting the potential advantage of early, biomarker-guided biologic therapy.

Furthermore, our patient achieved complete serologic remission without the use of corticosteroids or additional immunosuppressive agents, whereas the patient in the report by Vanthuyne et al ultimately required pulse corticosteroids, rituximab, and escalation to 300 mg of mepolizumab. These divergent outcomes suggest that timely escalation to the EGPA-indicated dose, guided by even subtle biomarker trends, may help prevent clinical relapse in selected patients. This proactive, biomarker-guided approach may reflect an evolving paradigm in the management of ANCA-positive EGPA, thus shifting from reactive treatment to anticipatory modulation using targeted biologic agents.

In conclusion, this case highlights the potential of IL-5 blockade not only to suppress eosinophilic inflammation but also to modulate ANCA activity in the absence of corticosteroids. Mepolizumab may thus serve as an effective steroid-sparing strategy for maintaining immunologic quiescence and preventing vasculitic relapse in EGPA.

Ethics statement: In accordance with institutional guidelines, ethics approval was not required for this case report. Written informed consent for the report’s publication was obtained from the patient.

## Disclosure statement

Disclosure of potential conflict of interest: O. Matsuno has received lecture fees from 10.13039/100004330GlaxoSmithKline, 10.13039/100004325AstraZeneca, 10.13039/100008792Novartis Pharma, and Sanofi. The rest of the authors declare that they have no relevant conflicts of interest.
